# Understanding the Fundamentals of Microporosity Upgrading in Zeolites: Increasing Diffusion and Catalytic Performances

**DOI:** 10.1002/advs.202100001

**Published:** 2021-07-04

**Authors:** Zhengxing Qin, Shu Zeng, Georgian Melinte, Tomáš Bučko, Michael Badawi, Yanfeng Shen, Jean‐Pierre Gilson, Ovidiu Ersen, Yingxu Wei, Zhongmin Liu, Xinmei Liu, Zifeng Yan, Shutao Xu, Valentin Valtchev, Svetlana Mintova

**Affiliations:** ^1^ State Key Laboratory of Heavy Oil Processing College of Chemical Engineering China University of Petroleum (East China) Qingdao 266580 China; ^2^ National Engineering Laboratory for Methanol to Olefins Dalian National Laboratory for Clean Energy iChEM (Collaborative Innovation Center of Chemistry for Energy Materials) State Key Laboratory of Catalysis Dalian Institute of Chemical Physics Chinese Academy of Sciences Dalian 116023 China; ^3^ University of Chinese Academy of Sciences Beijing 100049 China; ^4^ Institut de Physique et Chimie des Matériaux de Strasbourg UMR 7504 CNRS Université de Strasbourg 23 rue du Loess BP 43 Strasbourg F‐67034 France; ^5^ Department of Physical and Theoretical Chemistry Faculty of Natural Sciences Comenius University in Bratislava Ilkovičova 6 Bratislava SK‐84215 Slovakia; ^6^ Institute of Inorganic Chemistry Slovak Academy of Sciences Dúbravská cesta 9 Bratislava SK‐84236 Slovakia; ^7^ Laboratoire de Physique et Chimie Théoriques UMR 7019 CNRS – Université de Lorraine Nancy F‐54000 France; ^8^ Normandie Univ, ENSICAEN, UNICAEN, CNRS Laboratoire Catalyse et Spectrochimie 6 Boulevard Maréchal Juin Caen 14050 France

**Keywords:** density functional theory (DFT), HP ^129^Xe NMR, microporosity upgrading, molecular diffusion, zeolites

## Abstract

Hierarchical zeolites are regarded as promising catalysts due to their well‐developed porosity, increased accessible surface area, and minimal diffusion constraints. Thus far, the focus has been on the creation of mesopores in zeolites, however, little is known about a microporosity upgrading and its effect on the diffusion and catalytic performance. Here the authors show that the “birth” of mesopore formation in faujasite (FAU) type zeolite starts by removing framework T atoms from the sodalite (SOD) cages followed by propagation throughout the crystals. This is evidenced by following the diffusion of xenon (Xe) in the mesoporous FAU zeolite prepared by unbiased leaching with NH_4_F in comparison to the pristine FAU zeolite. A new diffusion pathway for the Xe in the mesoporous zeolite is proposed. Xenon first penetrates through the opened SOD cages and then diffuses to supercages of the mesoporous zeolite. Density functional theory (DFT) calculations indicate that Xe diffusion between SOD cage and supercage occurs only in hierarchical FAU structure with defect‐contained six‐member‐ring separating these two types of cages. The catalytic performance of the mesoporous FAU zeolite further indicates that the upgraded microporosity facilitates the intracrystalline molecular traffic and increases the catalytic performance.

## Graphical abstract

While the focus currently is on the creation of mesopores in zeolites, the microporosity upgrading is rarely considered. The authors report on the fundamentals of such a microporosity upgrading in zeolites and its impact on the molecular diffusion and catalyst performance using hyperpolarized ^129^Xe nuclear magnetic resonance (NMR) spectroscopy supported by electron tomography and density functional theory calculations.

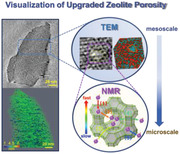

## 1 Introduction

Zeolites are crystalline aluminosilicates with unique framework topologies and adjustable acid properties widely used in catalysis, adsorption, and separation. The post‐synthesis removal of framework atoms and the associated formation of mesopores, that is, the creation of “highways” is already widely used to improve the intra‐crystalline molecular traffic of reactants and products.^[^ ^1^ ^]^ Recently, significant improvements in the design, synthesis, characterization, and use of these hierarchical zeolites were made.^[^ ^2^, ^3^, ^4^, ^5^, ^6^ ^]^ Despite such efforts, many important issues still need to be addressed. For instance, the mechanism of mesopore formation is not yet fully understood on a micro‐scale.^[^ ^7^ ^]^ Although various microscopy techniques enable to visualize mesoporosity at different length scales,^[^ ^8^, ^9^ ^]^ the initial step of their formation, that is, the removal of framework atoms and its consequences, is not yet fully understood.^[^ ^10^ ^]^

In addition, the enhanced diffusion and catalytic performances of hierarchical zeolites are currently associated with improved mass transfer due to the presence of mesopores, which increase the accessibility of reactants to active sites located in the microporosity.^[^ ^11^ ^]^ However, a quantitative description of the connectivity between the native micropores and the added mesopores required further attention.^[^ ^12^ ^]^ The size and shape of the zeolite micropores determine the shape‐selectivity of zeolite catalysts and are dramatically affected by subtle changes of such microporosity.^[^ ^13^, ^14^ ^]^ Considering the significant number of framework‐atom‐detachment events during the formation of hierarchical zeolites and the associated subtle changes in window size and shape of their micropores, referred to as microporosity upgrading, a noticeable impact on the diffusion and reaction path and product selectivity is expected.

In the present work, the connectivity between the micro‐ and meso‐pores in faujasite (FAU) zeolites is studied by hyperpolarized (HP) ^129^Xe nuclear magnetic resonance (NMR) spectroscopy and high resolution transmission electron microscopy (HRTEM). To that end, a series of hierarchical Y zeolites (FAU‐type framework structure, hereinafter referred as FY) are prepared and compared to the parent zeolite (hereinafter referred as PY). The hierarchical zeolites are prepared by the recently disclosed unbiased leaching of the parent zeolite with NH_4_F, producing derivatives with an identical Si/Al ratio.^[^ ^15^ ^]^ Further the diffusion of Xe in the hierarchical zeolites is studied by density functional theory (DFT). In particular the effect of point defects in the six‐membered rings (6MR), separating the sodalite (SOD) cages from the supercages, on the diffusion energy barrier is computed.

## 2 Results and Discussion

The crystallinity of all zeolite samples, parent and hierarchical, remains unchanged as shown by X‐ray diffraction (XRD), Figure S1, Supporting Information. The chemical composition of the samples (Si/Al ratio) is identical as determined by ^29^Si magic angle spinning (MAS) NMR (framework) and inductively coupled plasma atomic emission spectrometry (ICP‐AES) (bulk), Table S1, Supporting Information. Nitrogen physisorption results highlight a gradual formation of mesopores in the zeolite samples subjected to NH_4_F etching (FY series), Figures S2–S3 and Table S1, Supporting Information.

### 2.1 Microporosity Upgrading in Zeolites Revealed by Electron Tomography

**Figure 1:**
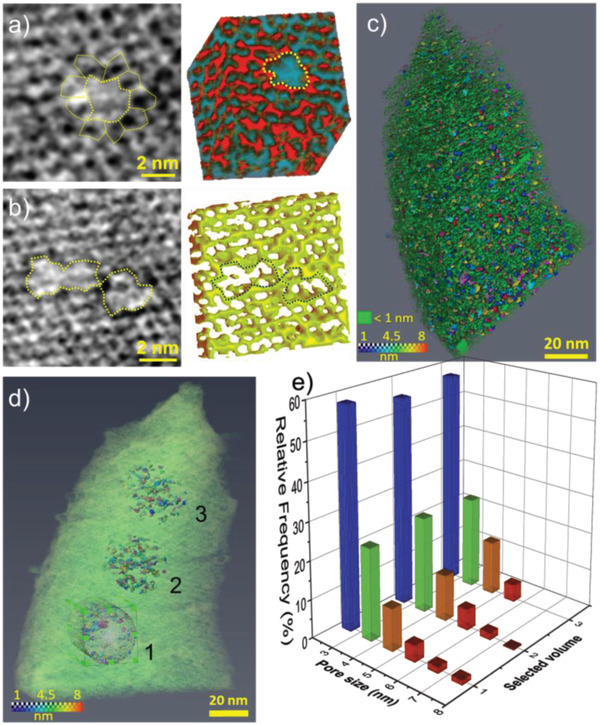
Electron tomography (3D‐TEM) of hierarchical zeolite FY20. a) High‐resolution view of a mesopore created via the merging of few microporous cages (left) and 3D representation of a mesopore and the surrounding in the zeolite crystal (right). b) High‐resolution view of a pair of three mesopores merged (left) and 3D representation of mesopores and their surrounding in a zeolite crystal (right). c) 3D reconstruction image of the FY‐20 zeolite visualizing the micro‐cages with a size of 0.7–1.5 nm (green frame) and larger mesopores (RGB colored). d) 3D reconstruction image containing three cylindrical volumes used for quantification of the porous network. e) Pore size distribution calculated from three distinct regions of the zeolite crystal presented in Figure 1d.

The dynamic evolution of the micro‐ and meso‐porosity of FY zeolites can be revealed by electron tomography (3D‐TEM), but 3D imaging of pores smaller than 2 nm is a difficult challenge for micron‐sized zeolite crystals. Therefore, thin specimens of ≈40 nm (Figure S4a, Supporting Information) are prepared by ultramicrotomy of hierarchical FY‐20 zeolite (Table S1, Supporting Information). The tomographic reconstruction highlights evenly distributed small mesopores with a diameter of 2–5 nm located in the FY‐2 zeolite (Figure S4b,c, Supporting Information). The high‐resolution 3D reconstruction shows some of the zeolite cages and their merging resulting in the formation of larger mesopores. The formation of such mesopores may be due to the etching of the walls of the cages that extends around point defects in the zeolite framework (**Figure** **1a**). The enlargement of the mesopores seems to appear when two or multiple mesopores merge due to the close proximity (Figure 1b). The segmentation of the 3D reconstruction reveals interconnected cages (0.7–1.5 nm, see the green frame of Figure 1c), homogenously punctured by larger mesopores (RGB colored, Figure 1c). Such high‐resolution reconstruction highlights the boundary between two zeolite nanograins with different crystallographic orientations (Figure S4d, Supporting Information). Along the 3D interface between the two zeolite grains, the concentration of mesopores increases substantially. This agrees with our previous findings highlighting the preferential formation of mesopores along defective sites.^[^ ^15^ ^]^

To quantify the porous networks, three cylindrical volumes with no contact with the zeolite's upper and lower surfaces are evaluated (Figure 1d). This approach avoids the introduction of errors arising from the sensitive step of surface segmentation. The quantification results based on the evaluation of the three volumes show that the mesopores alone occupy a fraction of ≈6% of the total zeolite space, while the primary micropores reach a fraction of around 25%. Such a micro‐ to mesopore volume ratio of 4 is in good agreement with the N_2_ sorption data (Table S1, Supporting Information). The mesopore size distribution determined from the selected volumes (Figure 1e) shows that more than 55% of the secondary porosity consists of pores with a diameter of 2–3 nm. The pores with a diameter of 3–4 nm and 4–5 nm account for ≈24% and 11–14%, respectively. Several pores larger than 5 nm are also present and formed by merging adjacent mesopores depicted in Figure 1b. This quantification is in line with the pore size distribution derived from nitrogen physisorption measurements (Figure S3 and Table S1, Supporting Information).

**Figure 2:**
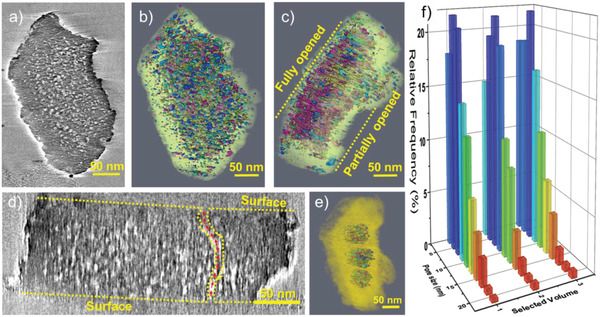
3D‐TEM pictures of hierarchical zeolite FY60. a) 3D‐TEM reconstruction of the FY60 zeolite crystal. b,c) 3D distribution of the mesoporous network of the FY60 zeolite crystal (Color label based on the spatial distribution and not the pore size as the porous network is highly connected. For a color coded distribution see Figure S5, Supporting Information). d) 3D‐TEM reconstruction of the FY60 zeolite crystal showing the interconnectivity of the mesopores from one to the other surfaces. e) 3D reconstruction image containing three isolated internal volumes. f) Pore size distribution calculated from three distinct regions of the zeolite crystal presented in Figure 2e.

The 3D‐TEM image of the FY60 hierarchical zeolite, with a lower micropore volume than the parent PY (Table S1, Supporting Information) is pictured in **Figure** **2**. The 3D reconstruction shows a dense network of isolated and interconnected mesopores (Figure 2a,b). A well‐connected network of mesopores extends from one external surface through the center of the zeolite crystal to the opposite surface (Figure 2c,d). The 3D‐TEM quantitative analysis indicates that around 90% of the mesopores are fully connected. The isolated mesopores, or those with double and triple connections, represent ≈10% of the total amount of mesopores. The quantification of the pore size distribution using three selected internal regions (Figure 2e) shows that most of the mesopores are in the 2–12 nm range (Figure 2f). The 2–4 nm mesopores account for ≈17% of the total mesoporosity. Such quantification, however, does not differentiate between connected and isolated mesopores. A modified segmentation of the pores, to take into account the individual pores even when they merge, shows that the mesopores with a size of 2–3 nm represent around 80% of the total mesoporous network. This quantification is confirmed by the pore size distribution analysis based on N_2_ physisorption measurements (Figure S3, Supporting Information).

The important finding is that the highest density of mesopores in the 2–3 nm range, that is, more than 55% of the total amount of mesopores is found in the FY20 zeolite sample. This suggests a possible merging of two FAU supercages,^[^ ^15^ ^]^ and the formation of mesopores by a continuous removal of framework atoms starting from the SOD cage between neighboring supercages. The “birth” of mesopores in the FAU zeolite starting with the possible removal of framework T atoms from the SOD cages was further investigated using advanced HP ^129^Xe NMR spectroscopy and supported by DFT calculations. The spatial distribution of the mesopores indicates that cages are opened in the entire volume of the zeolite crystals, and the process progresses during the etching process (sample FY60, Figure 2). As a result, while the existing mesopores increase in size and interconnectivity, new SOD cages are continuously opened, leading to the formation of new mesopores. The cage opening process leads to intermediate products with enhanced pore volume and preserved microporous characteristics.^[^ ^15^ ^]^ Additionally, the defects may favor mesopores formation (Figure S4d, Supporting Information) as reported before.^[^ ^15^ ^]^ Defects may control the dissolution preference of zeolite crystals. The FAU zeolite crystals investigated here behave differently from the MFI and mordenite (MOR) zeolites that followed a “reverse layer‐by‐layer” dissolution mechanism starting from outside micropores.^[^ ^16^, ^17^ ^]^

Based on the above results, we suggest that the enlarged supercages and the newly‐developed mesopores are formed as a unique interface between the mesoporous voids and the remaining micropores of the zeolite phase. Such a new porous network is likely to significantly affect the diffusion and the catalytic performance of these hierarchical zeolites.

### 2.2 Microporosity Upgrading in Zeolites Tracked by HP ^129^Xe NMR Spectroscopy

**Figure 3:**
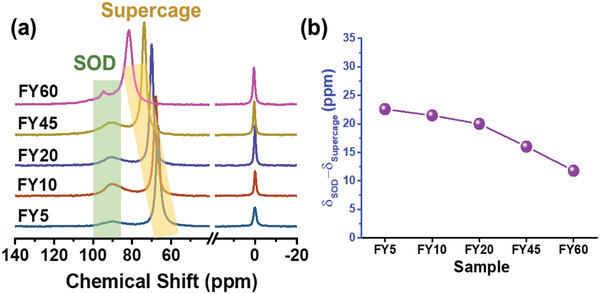
1D HP ^129^Xe spectra of hierarchical zeolites. a) HP ^129^Xe spectra of hierarchical zeolites (FY5–FY60) at 253 K; the green background highlights xenon located in opened SOD cages, the yellow background indicates xenon in supercages, the chemical shift at 0 ppm represents free xenon in gas phase. b) Difference in chemical shifts between the peaks associated with the xenon in the SOD cages and supercages in the hierarchical zeolites.

The diffusion pathway in the hierarchical FAU zeolites was investigated by HP ^129^Xe NMR spectroscopy. ^129^Xe NMR is a powerful technique to probe the porosity at the molecular level.^[^ ^18^, ^19^, ^20^ ^]^ For instance, we showed earlier that Xe cannot enter SOD cages in a parent zeolite Y due to its large kinetic diameter.^[^ ^15^ ^]^ In the current study, the HP ^129^Xe NMR spectra of all hierarchical zeolites were collected at 253 K (**Figure** **3**). All spectra display a peak at ≈90 ppm attributed to xenon located in opened SOD cages.^[^ ^15^ ^]^ The difference in chemical shift of this peak and the one attributed to Xe in the supercages (67.5–81.8 ppm) narrows from the FY5 to the FY60 samples by 23 ppm to 12 ppm, respectively (Figure 3a,b). HP ^129^Xe NMR provides strong evidence that the exchange of Xe is faster between the opened SOD cages and supercages as a result of the continuous opening of the SOD cages and formation of mesopores. The variable temperature (VT) HP ^129^Xe NMR experiments on the hierarchical zeolites were also performed with the attempt to distinguish the mesopores from the micropores (Figure S6, Supporting Information). Due to the quick exchange of Xe between the mesopores and micropores under the experimental conditions applied no a distinct conclusion was made. Therefore experiments at lower temperatures (153 K) should be considered in the future but yet this is beyond the temperature limit allowed by our equipment set‐up.

**Figure 4:**
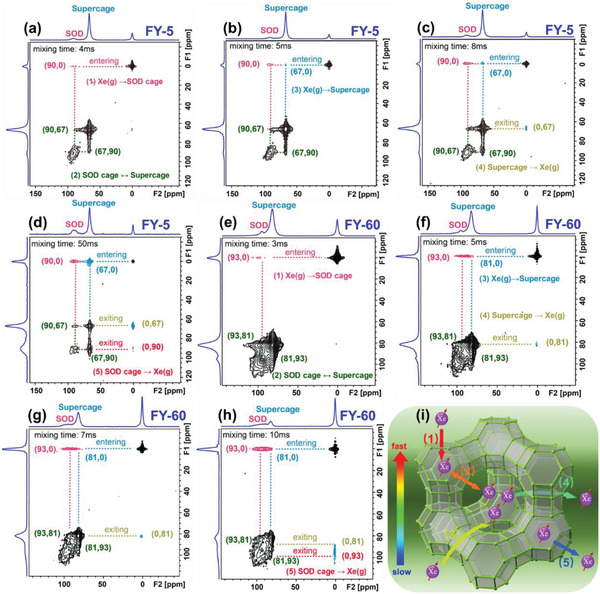
Diffusion of xenon in hierarchical zeolites. HP ^129^Xe EXSY NMR spectra xenon on the FY5 zeolite at different mixing times: a) 4 ms. b) 5 ms. c) 8 ms. d) 50 ms. HP ^129^Xe EXSY NMR spectra of xenon on the FY60 zeolite at different mixing times: e) 3 ms. f) 5 ms. g) 7 ms. h) 10 ms. All spectra are recorded at 253 K. i) Schematic representation of the Xe diffusion paths in hierarchical zeolites: 1) adsorption of Xe from the gas phase in opened SOD cages, 2) simultaneous diffusion of Xe in SOD cages and exchange with supercages, 3) diffusion of Xe from the gas phase to the supercages, 4) subsequent diffusion of Xe out of supercages, and 5) desorption of Xe from SOD cages.

The kinetics of xenon exchange between the opened SOD cages and supercages is further studied by HP ^129^Xe 2D‐Exchange spectroscopy (EXSY) NMR at 253 K. The continuous flow HP ^129^Xe 2D‐EXSY NMR experiment may provide further information about the adsorption sites and pore inter‐connectivity in different adsorption regions.^[^ ^21^, ^22^ ^]^ The HP xenon quickly loses its polarization after interacting with the zeolite pore walls resulting in weakening of the signal (depolarization). The correlation peak observed in the 2D HP ^129^Xe EXSY NMR spectra primarily occurs due to the adjacent micropores. On sample FY5, a cross‐peak occurs at (90, 0) (F2, F1) ppm at a mixing time of 4 ms (**Figure** **4a**). This evidences that the gaseous xenon enters the SOD cages. Two other exchange peaks occur at (90, 67) ppm and (67, 90) ppm corresponding to xenon exchange between the SOD cages and supercages taking place almost simultaneously in both directions. At mixing time of 5 ms, a cross peak occurs at (67, 0) ppm which is assigned to the diffusion of gaseous xenon in the supercages of the zeolite sample (Figure 4b). The diffusion via supercages is the only energetically feasible path for perfect FAU defect‐free zeolite crystals. With increasing the mixing time to 8 ms, a cross peak occurs at (0, 67) ppm, which is attributed to the desorption of xenon confined in supercages to the gas phase (Figure 4c). A further increase of the mixing time to 50 ms provides a new cross peak at (0, 90) ppm attributed to xenon's desorption from the SOD cages to the gas phase (Figure 4d). These 2D HP ^129^Xe EXSY NMR experiments provide further proof that some SOD cages are opened and accessible for the xenon, enabling the fast exchange of xenon between opened SOD cages and supercages (Figure 4a). Moreover, by using different mixing times, the diffusion path of xenon in the microporous volume of the FY5 hierarchical zeolite is monitored. Interestingly, gaseous xenon first adsorbs in the opened SOD cages, and then diffuses to the supercages (Figure 4). For the hierarchical FY45 and FY60 zeolite samples with decreased micropore volume, increased mesopore volume (Table S1, Supporting Information), and improved mesopore accessibility (Figure 2), the diffusion of xenon inside zeolite cages is even faster (Figure 4e–h and Figure S7, Supporting Information). In both hierarchical FY45 (Figure S7, Supporting Information) and FY60 (Figure 4e–h) zeolite samples, the cross‐peaks corresponding to xenon adsorption in the SOD cages from gas phase and exchange of xenon between SOD and supercages, respectively occur at a mixing time of 3 ms (Figure 4e and Figure S7a, Supporting Information). In comparison, the xenon exchange between supercages and the gas phase for sample FY60 occurs at 5 ms mixing time (Figure 4f and Figure S7b, Supporting Information). All signals in the spectra at 20 ms mixing time are reported in Figure 4h and Figure S7c, Supporting Information. In the hierarchical FY60 zeolite, the desorption of xenon from the SOD cages to the gas phase occurs at the mixing time as short as 10 ms (Figure 4h).

The results discussed above demonstrate that an increased intraparticle diffusion is provided by opening of the SOD cages and enhancing the mesoporosity of the hierarchical FAU zeolite. The 2D HP ^129^Xe EXSY NMR spectra show that opening SOD cages not only free a hitherto unreachable space,^[^ ^15^ ^]^ but also substantially change the diffusion paths in the hierarchical FAU zeolite (Figure 4i). The current methodology applied to evaluate porosity of zeolites could oversimplify the contribution of diffusion limitations especially of concerns in catalysis and gas separation applications. The results show that even in the case of decreased micropore volume of zeolites, the micropores accessibility may change substantially via the mesopores surrounding and improved inter‐connectivity and small changes of the pore (cage) openings.

### 2.3 Microporosity Upgrading in Zeolites Revealed by DFT Modeling

Xe diffusion in the hierarchical FAU zeolites is modeled by DFT calculations. The FAU 3D structure consisting of SOD cages (diameter 6.6 Å) and supercages (diameter of 12.4 Å) are interconnected by hexagonal prisms of 2.3 Å (D6R); the supercages have a diameter of 7.4 Å. The pure silica FAU framework is represented by the face‐centered cubic cell (space group Fd3m),^[^ ^23^ ^]^ and a lattice parameter of 25.028 Å for a unit cell consisting of 576 atoms (Si_192_O_384_).^[^ ^24^, ^25^ ^]^ In the current calculations, a primitive rhombohedral cell containing 144 atoms is used (Figure S8, Supporting Information). The primitive cell of the FAU contains two supercages and eight hexagonal windows connecting the SOD with the supercage. In order to examine a possible reduction of diffusion barrier due to the presence of the Brønsted Acid (BA) sites or defects created upon removal of T sites from the 6MR between the SOD cage and the supercage, seven structural models were considered: the pure siliceous structure (model A), a structure with one BA site (model B), with three BA sites (model C), with one defect (model D), two defects (model E), three defects (model F) and four defects (model G). Models A–C are displayed in Figure S9, Supporting Information and models D–G in Figure S10, Supporting Information.

**Figure 5:**
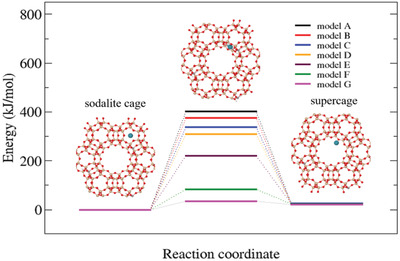
Potential energy profiles for diffusion of Xe from SOD cages to supercages in hierarchical zeolites. Seven models are considered: pure siliceous FAU type framework structure (model A), FAU type framework structure with one (models B) and three (model C) Brønsted acid sites; pure siliceous FAU type structures with one (model D), two (model E), three (model F) and four (model G) silanol nest defects.

**Figure****5** shows the potential energy barriers computed for the Xe diffusing from the SOD cages to the supercages. In the case of the pure siliceous FAU structure (model A), the diffusion barrier is as high as 402 kJ mol^−1^. As evident from Figure S9, Supporting Information, the passage through the 6MR separating the two voids is linked with a significant structural deformation of the ring caused by the repulsive interaction between the Xe and the framework atoms. One can therefore expect that increasing the size of the 6MR via the substitution of Si for Al (the Al─O bond is 0.1–0.3 Å longer than the Si─O bond, Figure S9, Supporting Information) can lead to stabilization of the transition state (TS) and hence decrease the diffusion barrier. This is indeed the case, one Al substitution decreases the diffusion barrier to 376 kJ mol^−1^, while three substitutions lead to an even more significant reduction to 339 kJ mol^−1^ (Figure 5). Since the diffusion barrier remains very high even in the latter case and the 6MR cannot accept more Al sites without violating the Löwenstein rule,^[^ ^24^ ^]^ we conclude that the Xe diffusion between the SOD cage and the supercage of parent FAU with a pure microporous structure is not kinetically feasible. Further, the FAU structure containing silanol nest defects created by replacing one (model D), two (model E), three (model F), and four (model G) neighboring tetrahedral sites from the 6MR separating the SOD cage from the supercage and terminating the dangling Si─O bonds by H atoms were considered (Figure S10, Supporting Information). In this way, the size and flexibility of the 6MR were increased since the strong covalent bonds between the tetrahedral and oxygen atoms were replaced by much weaker H‐bonds. As shown in Figure 5, the introduction of silanol nests leads to a significant reduction of the diffusion barrier from 402 kJ mol^−1^ in the pure defect‐free FAU zeolite to 83 kJ mol^−1^ in the structure containing three silanol nests. More importantly, the FAU zeolite structure containing four silanol defects further reduced the diffusion barrier to 34 kJ mol^−1^. The DFT results support the experimentally observed Xe diffusion that takes place in hierarchical FAU structure with 6MR containing defects separating the SOD cages from the supercages. Regardless of the particular structural model used in our simulations, the Xe adsorbed in the SOD cage was ≈20 kJ mol^−1^ lower in energy than in the supercage. This result is a direct consequence of a more general phenomenon of increasing van der Waals (vdW) stabilization with decreasing volume of confined voids of zeolite.^[^ ^26^ ^]^ Thus one can expect that the SOD cages will be occupied before the supercages if both are available and accessible for the Xe adsorption.

### 2.4 Microporosity Upgrading in Zeolites Changed the Catalyst Performance

The catalytic performance of the hierarchical FAU zeolites is assessed in two reactions: 1,3,5‐triisopropylbenzene (TiPBz) dealkylation and n‐octane (n‐C_8_) hydroconversion. In the dealkylation of TiPBz, the parent zeolite PY and its hierarchical derivative FY5 show identical conversions (Figure S11a, Supporting Information).^[^ ^15^ ^]^ The FY5 hierarchical zeolite sample with some SOD cages opened shows initially a lower 1,3‐diisopropylbenzene yield (Figure S11b, Supporting Information) but higher 1,4‐diisopropylbenzene (Figure S11c, Supporting Information) and 1,2‐diisopropylbenzene (Figure S11d, Supporting Information) yield for the bulkier isomer. During the dealkylation of TiPBz, 1,3‐diisopropylbenzene is the primary product, 1,4‐diisopropylbenzene and 1,2‐diisopropylbenzene are the secondary isomerized products derived from 1,3‐diisopropylbenzene. Apparently, the microporous FY5 zeolite favors the conversion of 1,3‐diisopropylbenzene with a kinetic diameter of 0.84 nm, which is still larger than 0.74 nm (the intrinsic micropore opening of zeolite Y). Meanwhile, it favors the formation of the secondary products including 1,2‐diisopropylbenzene, a bulkier secondary product than 1,3‐diisopropylbenzene. These evidently demonstrate that a subtle change in microporous properties of zeolites such as opened SOD cages, enlarged supercages, and micropore deformation may cause a substantial change in their catalytic performance. This observation is in line with their thermodynamic distribution.^[^ ^27^ ^]^ The hierarchical FY60 zeolite, with the lower micropore volume (Table S1, Supporting Information) and lower amount of acid sites (Figure S12, Supporting Information) shows a higher conversion in n‐C_8_ hydroconversion (Figure S11e,f, Supporting Information). These results further demonstrate that microporosity upgrading benefits the intracrystalline molecular traffic and increases the catalytic performance of zeolites.

## 3 Conclusion

The evolution of micropores and mesopores in hierarchical zeolites with the FAU‐type framework structure is monitored by 3D‐TEM and HP ^129^Xe NMR. The formation of mesopores in the FAU zeolite is found to be due to the unbiased removal of framework T atoms (T = Si or Al) by NH_4_F etching. The removal of the T atoms starts from the supercages, opening some SOD cages, and further allowing the connection of neighboring cages. Based on the Xe adsorption and diffusion study in the hierarchical FAU zeolite samples monitored by HP ^129^Xe NMR spectroscopy we revealed the increased intraparticle diffusion and a new diffusion pathway was proposed. Both the experimental and theoretical results concur that Xe first diffuses into the opened SOD cages before diffusing to the supercages of FAU zeolite. The superior catalytic performances are a direct result of the availability of more active sites and lower transport limitations in the hierarchical FAU zeolite crystals due to newly available diffusion pathways. As several industrially relevant zeolites contain cages (FAU, CHA, LTA, AEI…)that are often difficult to access even for small molecules (H_2_, NH_3_, CH_4_…), the crystal engineering described in this paper could unlock further potential in catalysis, separation by selective adsorption, capture and storage of molecules.

## 4 Experimental Section

### Materials and Characterization

A commercial Y zeolite (Y‐54 from UOP, Si/Al = 2.6) in its NH_4_‐form was used in this study (parent sample, PY). The fluoride medium etching was carried out in a 25 wt% NH_4_F (98.0%, Sigma Aldrich) aqueous solution with a liquid‐to‐solid ratio of 6. The treatment was performed under mechanical stirring and ultrasonic radiation (USC 600 TH, 45 kHz, VWR) in an ice bath (277 K) for different times (5–60 min). The resulting samples were denoted as FYX, with the suffix X represents the etching time in minutes, *X* = 5, 10, 20, 45, and 60 min. For example, FY5 represents the zeolite Y sample treated by NH_4_F for 5 min. The solid products after treatments were thoroughly washed and then dried.

XRD patterns of the parent and the treated samples were collected on a PANalytical X'Pert Pro diffractometer using Cu K*α* radiation (*λ* = 1.5418 Å, 45 kV, 40 mA) with a scanning step of 0.0167° s^−1^. The chemical composition of zeolite samples was analyzed by an inductively coupled plasma optical emission spectrometry (ICP, OPTIMA 4300 DV). Nitrogen physisorption analysis was carried out on a Micromeritics ASAP 2020 gas adsorption analyzer. Prior to analysis, the samples were degassed at 373 K for 1 h and 573 K for 10 h. The BET equation was used for the determination of the specific surface areas of the samples. The t‐plot method was used to determine the micropores volume and external surface areas. The BJH model was applied to the adsorption branch of the isotherms for the mesopore size distributions.

### Solid‐State NMR Measurements

HP ^129^Xe NMR experiments were conducted on a Bruker Avance III 400 MHz spectrometer using a home built continuous‐flow HP xenon delivery apparatus. Before the NMR measurements, the samples were dehydrated under vacuum at 693 K for 6 h, then transformed into a 5 mm quartz tube in an argon glove box. HP ^129^Xe was obtained with an optical pumping cell in the 50 Gauss field and the 60 W diode laser array (Coherent FAP‐System). The mixture gas consisting of 1% Xe, 1% N_2,_ and 98% He was delivered to the quartz NMR tube (5 mm) at the rate of 150 mL min^−1^. The resonance frequency of ^129^Xe was 110.7 MHz. 1D HP ^129^Xe NMR spectra were accumulated by a single pulse sequence with *π*/2 pulse width of 6 *μ*s and 2 s recycle delay. Variable temperature (VT) experiments were conducted with the temperature varying from 298 to 213 K. The *π*/2‐t1‐*π*/2‐tmix‐*π*/2‐t2 pulse sequence^[^ ^28^ ^]^ was used in 2D EXSY NMR experiment with the mixing time range of 1 to 100 ms under 253 K (Scheme S1, Supporting Information). The chemical shifts were referencedo gas phase xenon signal at 0 ppm. All the ^27^Al MAS NMR and ^29^Si MAS NMR experiments were performed on a Bruker Avance III 600 spectrometer equipped with a 4 mm H‐X double resonance WVT probe. The resonance frequencies for ^27^Al, ^29^Si were 156.4 and 119.2 MHz, respectively. The ^27^Al spectra were recorded using one pulse sequence with spinning rate of 12 kHz and chemical shifts were referenced to (NH_4_)Al(SO_4_)_2_·12H_2_O at −0.4 ppm. The ^27^Al MAS NMR experiments were conducted using the dehydrated sample. ^29^Si MAS NMR spectra were accumulated using high‐power proton decoupling sequence with spinning rate of 10 kHz, and chemical shifts were referenced to kaolinite at −91.5 ppm.

### TEM Measurements

TEM images were recorded using a JEOL 2100F microscope equipped with a high‐resolution objective lens pole piece at 200 kV. The tomography series were acquired between tilting angles of ±70° with a 2° Saxton scheme, and the subsequent series alignments were performed with the IMOD software using Au nanoparticles (5–7 nm) as fiducial markers. To resolve details at maximum resolution the SIRT algorithm implemented in fast software running on multicore computers, Tomo3D was used. The size of the TEM projections used for the reconstruction was 2k × 2k pixels.

### DFT Calculations

The periodic DFT calculations were performed using the Vienna Ab initio Simulation Package (VASP).^[^ ^29^, ^30^ ^]^ The PBE exchange‐correlation functional in the generalized gradient approximation (GGA) proposed by Perdew et al.^[^ ^31^ ^]^ was employed. The electron–ion interactions were described using the projector augmented plane wave (PAW) method of Blöchl^[^ ^32^ ^]^ as adapted by Kresse and Joubert.^[^ ^33^ ^]^ A planewave cutoff energy of 400 eV was used in all calculations. The Kohn–Sham equations were solved self‐consistently until the energy difference between cycles becomes lower than 10^−6^ eV. In order to describe the adsorption process in the zeolite with a good precision, dispersion interactions were taken into account. An atom‐pairwise D2 correction of Grimme^[^ ^34^, ^35^ ^]^ was used for this purpose, as applied previously to calculate a reasonable structure and energetics for zeolites.^[^ ^36^, ^37^, ^38^ ^]^ The atomic positions were optimized until all forces were smaller than 0.005 eV Å^−1^ per atom. A primitive rhombohedral cell of FAU with two supercages containing total of 144 atoms was used for the calculations (Figure S8, Supporting Information). The lattice parameters, obtained after relaxation of the atomic positions and the cell geometry at the PBE+D2 level were a = 17.34 Å and α = 60°. Considering the large size of the unit cell, the Brillouin zone sampling was realized using a single (Γ) k‐point. The transition state (TS) optimization was performed using the optimization engine GADGET.^[^ ^39^, ^40^ ^]^ In order to identify the stable configurations linked with TS via a common transformation path, the intrinsic reaction coordinate^[^ ^41^, ^42^ ^]^ for the forward and backward reaction steps was identified using the damped velocity Verlet algorithm.^[^ ^43^ ^]^ The vibrational eigenspectra of all relaxed structures were checked to ensure that the given state corresponds to expected stationary point on the potential energy hypersurface.

### Catalytic Tests

The zeolite samples were tested in two catalytic reactions, the 1,3,5 tri‐isopropylbenzene dealkylation and n‐octane hydroconversion. The catalytic tests were carried out following the conditions reported before.^[^ ^15^ ^]^

## Conflict of Interest

The authors declare no conflict of interest.

## Supporting information

Supporting Information

## Data Availability

Research data are not shared.
