# Supplementary material for: Understanding the Fundamentals of Microporosity Upgrading in Zeolites: Increasing Diffusion and Catalytic Performances
Source: Adv Sci (Weinh). 2021 Jul 4;8(17):2100001. doi: 10.1002/advs.202100001 (PMC8425932; doi:10.1002/advs.202100001)
Supplement: Supplementary file 1 — Supporting Information [file ADVS-8-2100001-s001.pdf]

WILEY-VCH

© 2021 Wiley-VCH GmbH

ADVANCED SCIENCE
Open Access

Supporting Information

for *Adv. Sci.,* DOI: 10.1002/advs.202100001

Understanding the fundamentals of microporosity upgrading in zeolites: increasing diffusion and catalytic performances

Zhengxing Qin†, Shu Zeng†, Georgian Melinte, Tomáš Bučko, Michael Badawi, Yanfeng Shen, Jean-Pierre Gilson, Ovidiu Ersen, Yingxu Wei, Zhongmin Liu, Xinmei Liu, Zifeng Yan, Shutao Xu, Valentin Valtchev and Svetlana Mintova**

Supporting Information

Understanding the fundamentals of microporosity upgrading in zeolites: increasing diffusion and catalytic performances

Zhengxing Qin†, Shu Zeng†, Georgian Melinte, Tomáš Bučko, Michael Badawi, Yanfeng Shen, Jean-Pierre Gilson, Ovidiu Ersen, Yingxu Wei, Zhongmin Liu, Xinmei Liu, Zifeng Yan, Shutao Xu, Valentin Valtchev and Svetlana Mintova**

Table of Contents

Table S1.
Scheme S1.
Figure S1-S9
Reference

Table S1. N_2 physisorption analysis of parent (PY) and hierarchical (FY) zeolites, and their Si/Al ratio determined by NMR and ICP analyses.

Sample	Si/Al_{ICP}[a]	Si/Al_{NMR}[b]	S_{BET}[c]	V_{mic}[d]	S_{ext}[d]	V_{meso}[e]
		NH_4-type	m^2g^{-1}	cm^3g^{-1}	m^2g^{-1}	cm^3g^{-1}
PY	2.6	2.5	741	0.34	40	0.04
FY5	2.6	2.7	786	0.36	45	0.06
FY10	2.7	2.8	785	0.37	33	0.04
FY20	2.9	2.9	781	0.35	66	0.09
FY45	-	3.1	795	0.33	129	0.15
FY60	3.6	3.6	824	0.28	246	0.25

[a] ICP analysis. [b] NMR analysis. [c] BET surface area. [d] t-method; [e] $V_{meso} = V_{total} - V_{mic}$ (V_{total}: the volume adsorbed at $P/P_0 = 0.99$).

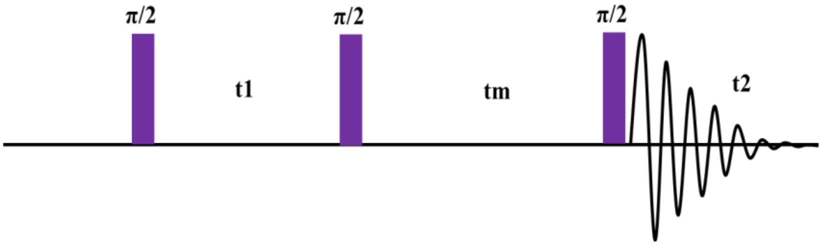

Scheme S1. 2D EXSY NMR pulse sequence used in this work.[1]

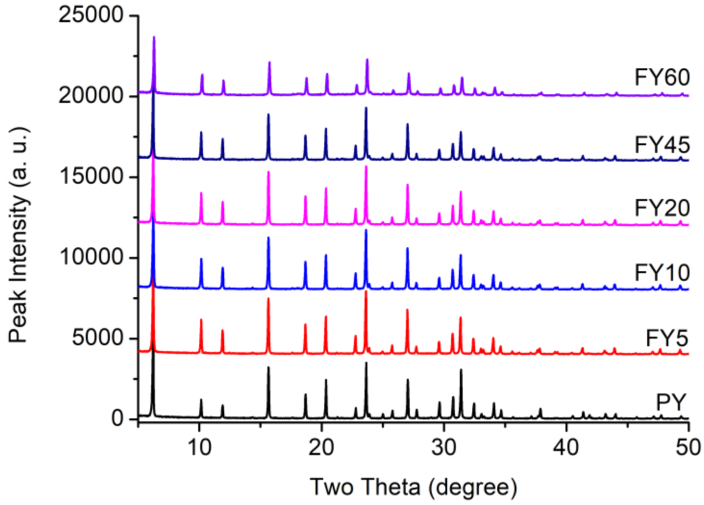

Figure S1. XRD patterns of the parent (PY) and hierarchical (FY5 - FY60) zeolites.

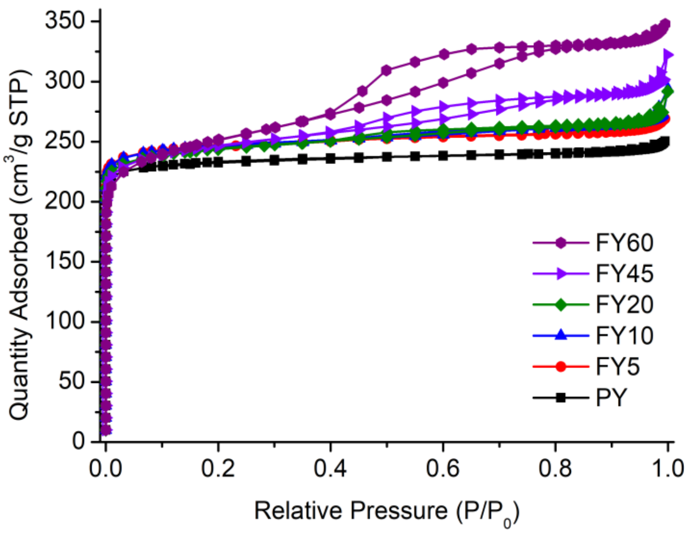

Figure S2. Nitrogen physisorption isotherms of the parent (PY) and hierarchical (FY5 - FY60) zeolites.

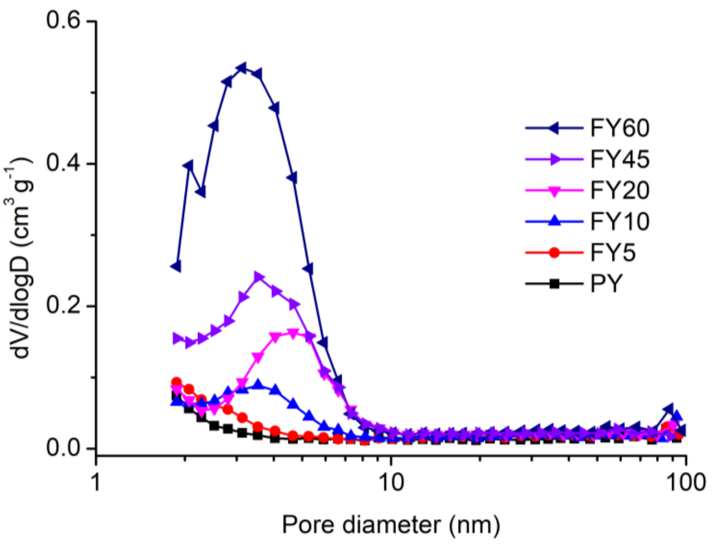

Figure S3. Pore size distributions of the parent (PY) and hierarchical (FY5 - FY60) zeolites derived from the adsorption branch of the isotherms.

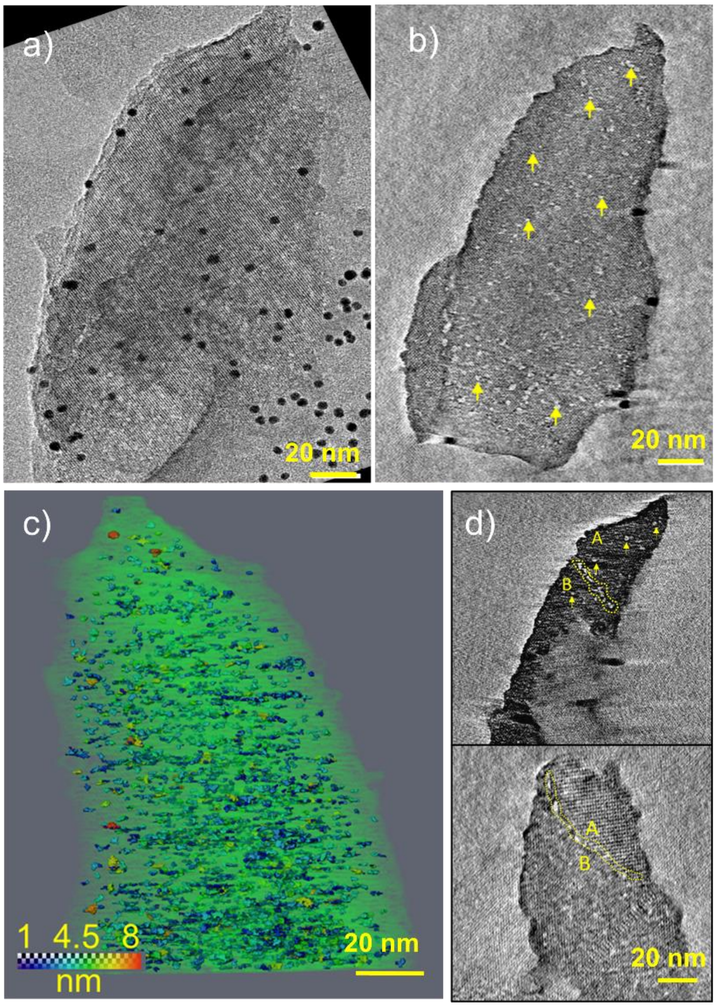

Figure S4. (a) TEM picture of hierarchical zeolite FY-20: 40 nm thick slice was prepared by ultramicrotomy. (b) 3D-TEM reconstruction of the FY20 zeolite; yellow arrows highlight the position of secondary mesopores. (c) Color coded (left corner) 3D mesoporous network of the hierarchical FY20 zeolite. (d) High-resolution reconstruction images viewing the boundary between two zeolite nanograins (A and B) with different crystallographic orientations.

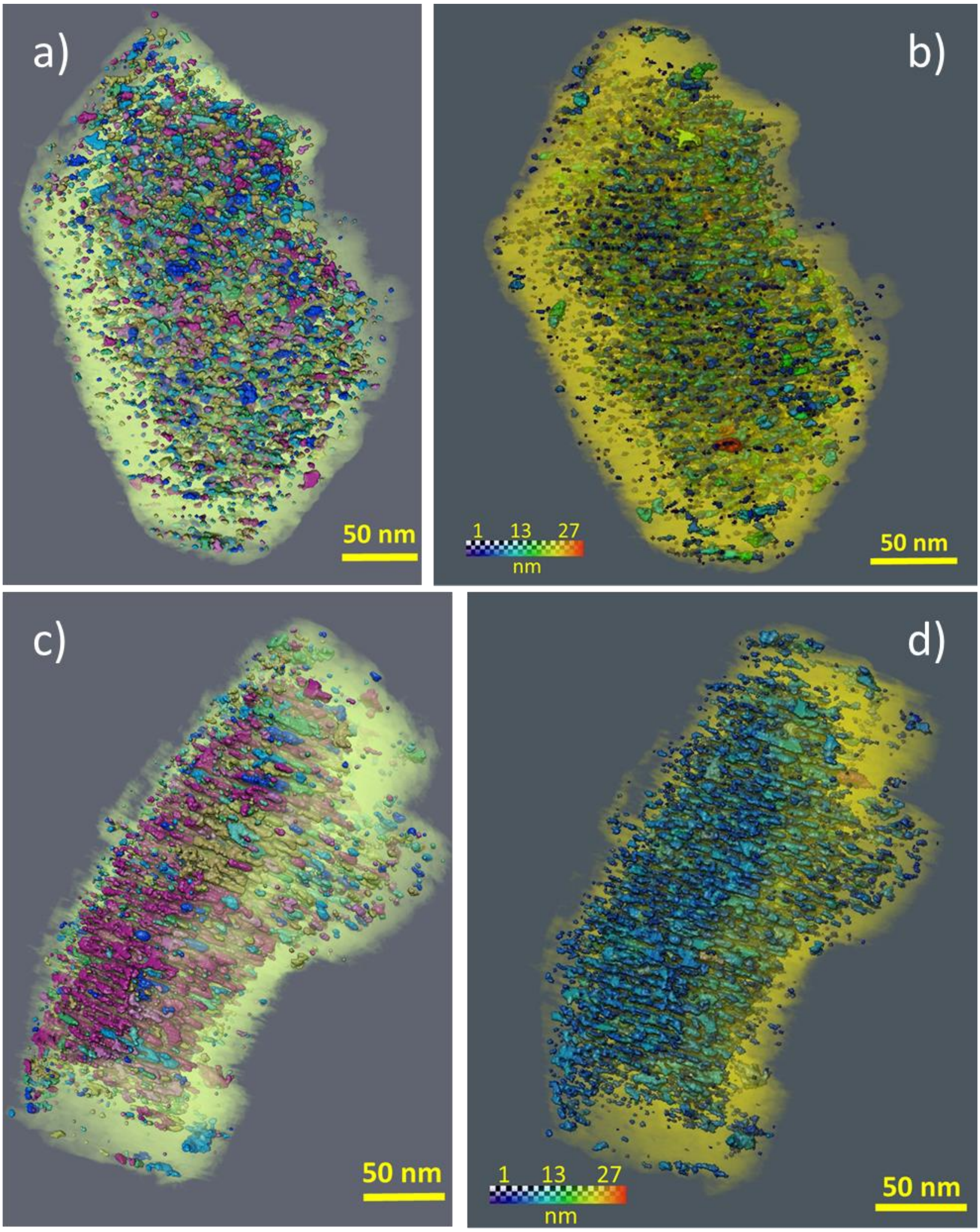

Figure S5. 3D-TEM pictures of hierarchical zeolite FY60. a) and c) 3D distribution of the mesoporous network of the FY60 zeolite crystal. Conventional color label based on the spatial distribution of the pores inside the reconstructed volume. b) and d) Color coded 3D distribution of the mesoporous network of the FY60 zeolite crystal based on individual pore size.

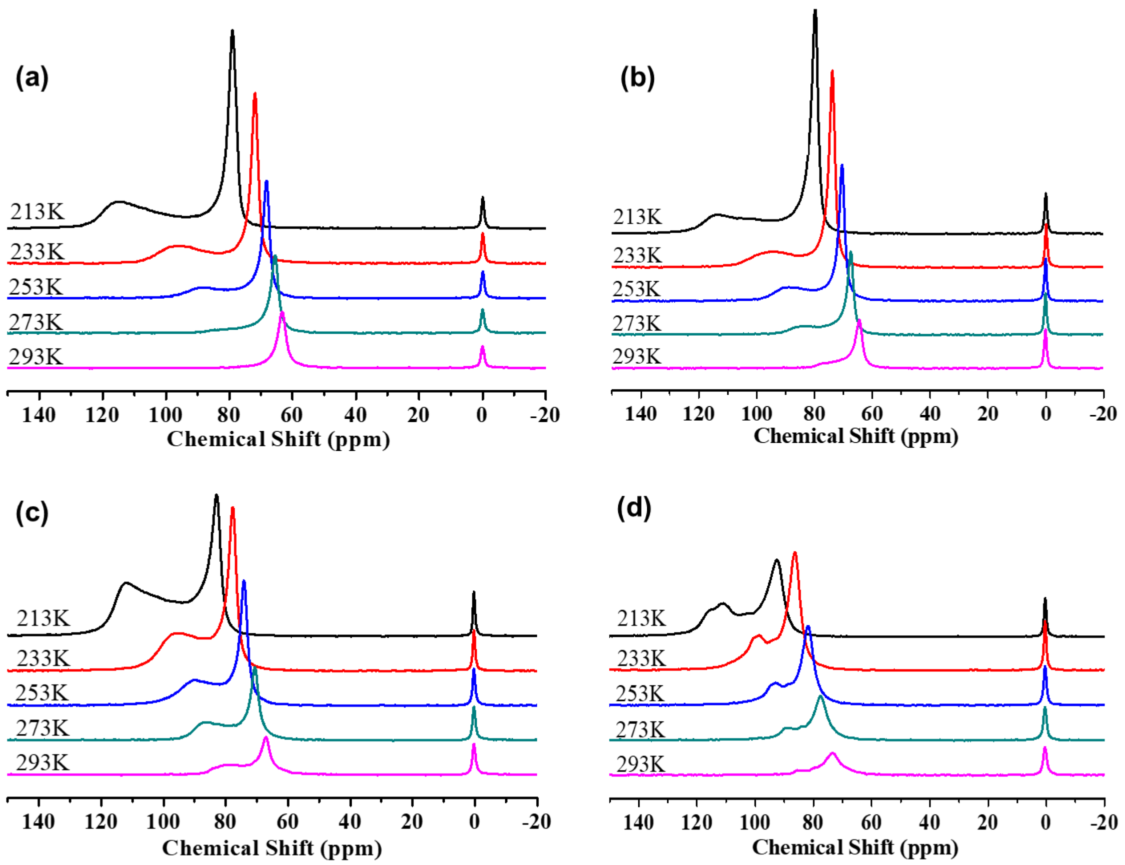

Figure S6. The HP ^{129}Xe NMR spectra of hierarchical zeolites at different temperatures: (a) FY10, (b) FY20, (c) FY45 and (d) FY 60.

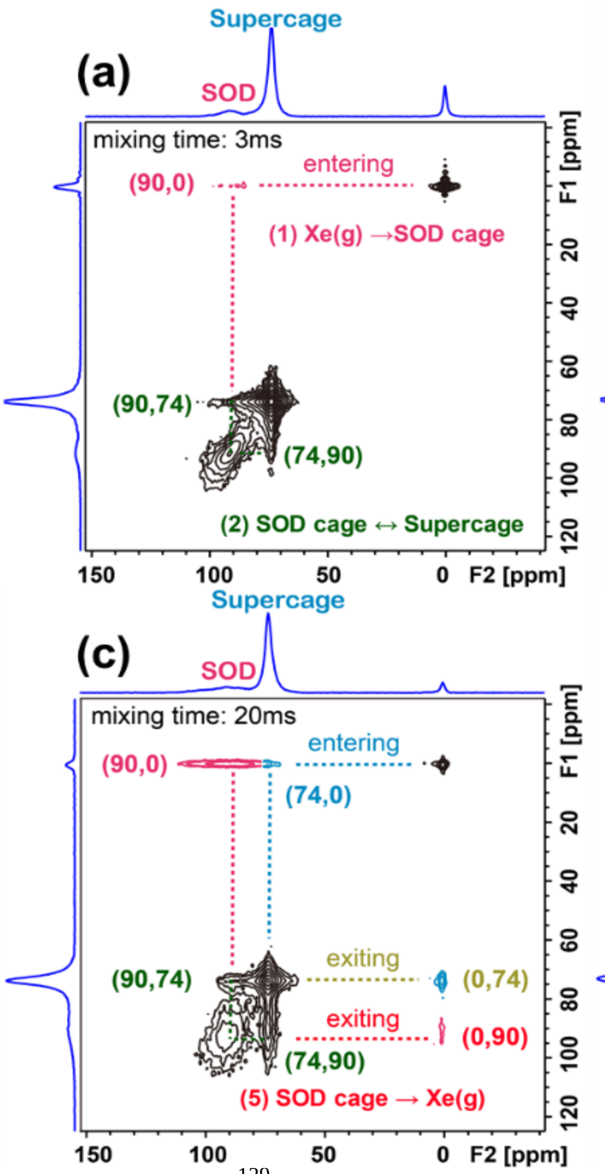

Figure S7. 2D HP ^{129}Xe EXSY NMR spectra of xenon on hierarchical zeolite FY45 at 253 K with mixing times of (a) 3 ms, (b) 5 ms, (c) 20 ms and (d) 50 ms.

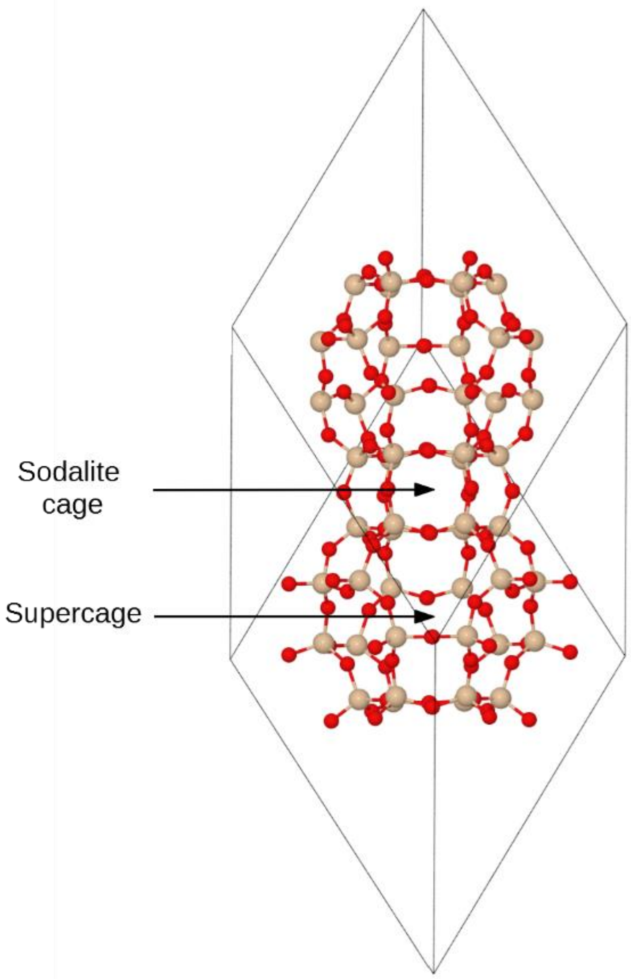

Figure S8. Primitive rhombohedral cell (144 atoms) employed for the DFT calculations. Color codes: O (red), Si (yellow).

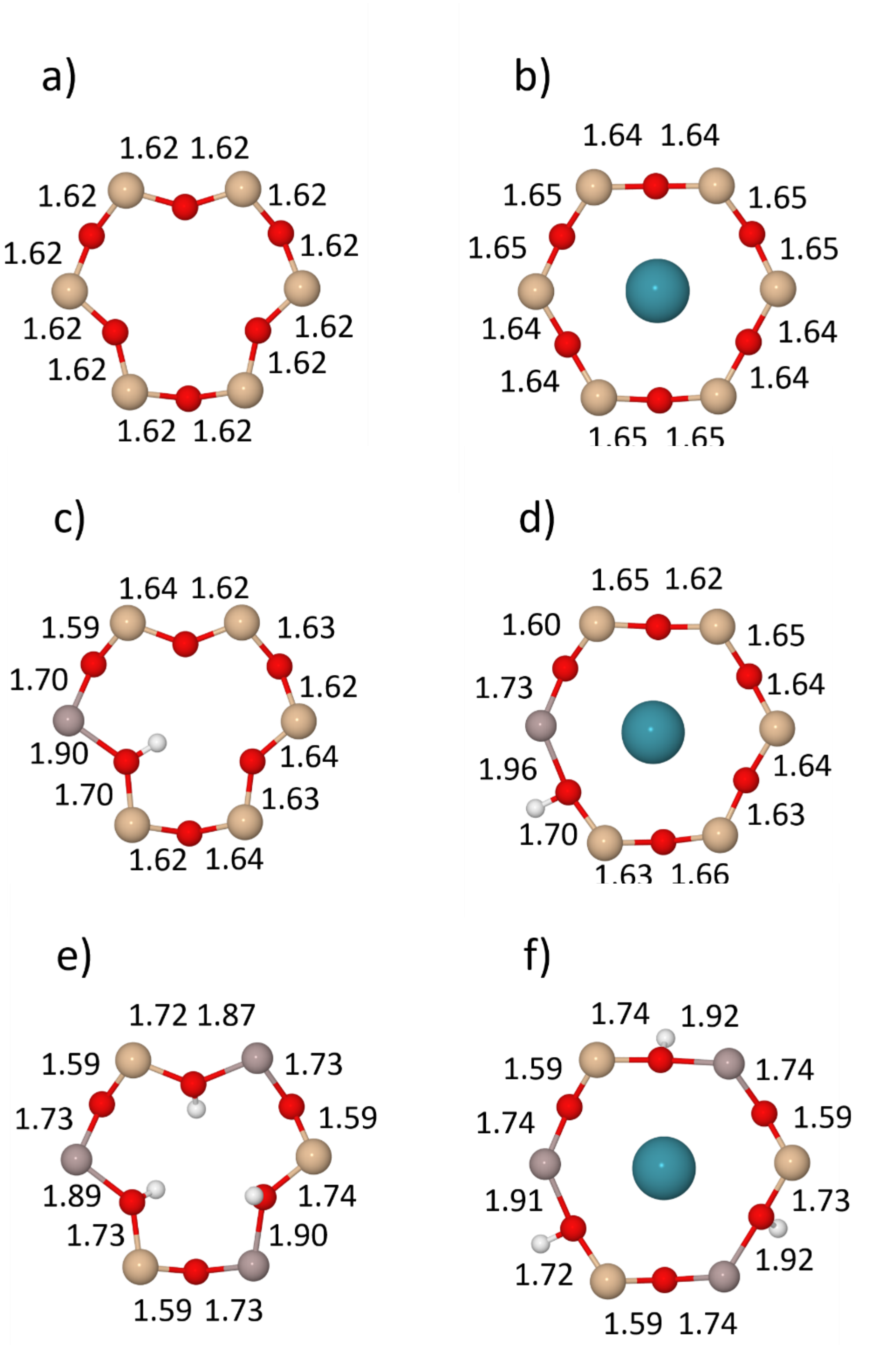

Figure S9. Detailed view of the six membered rings separating the sodalite cage from the supercage: (a, c, e) in stable configurations with Xe in the supercage and (b, d, f) in diffusion transition states. The three structural models considered: (a, b) model A: purely siliceous FAU zeolite, (c, d) model B: structure with one BA site on 6MR, and (e, f) model C: structure with three BA sites on 6MR. The lengths of the Si-O and Al-O bonds are given in Å.

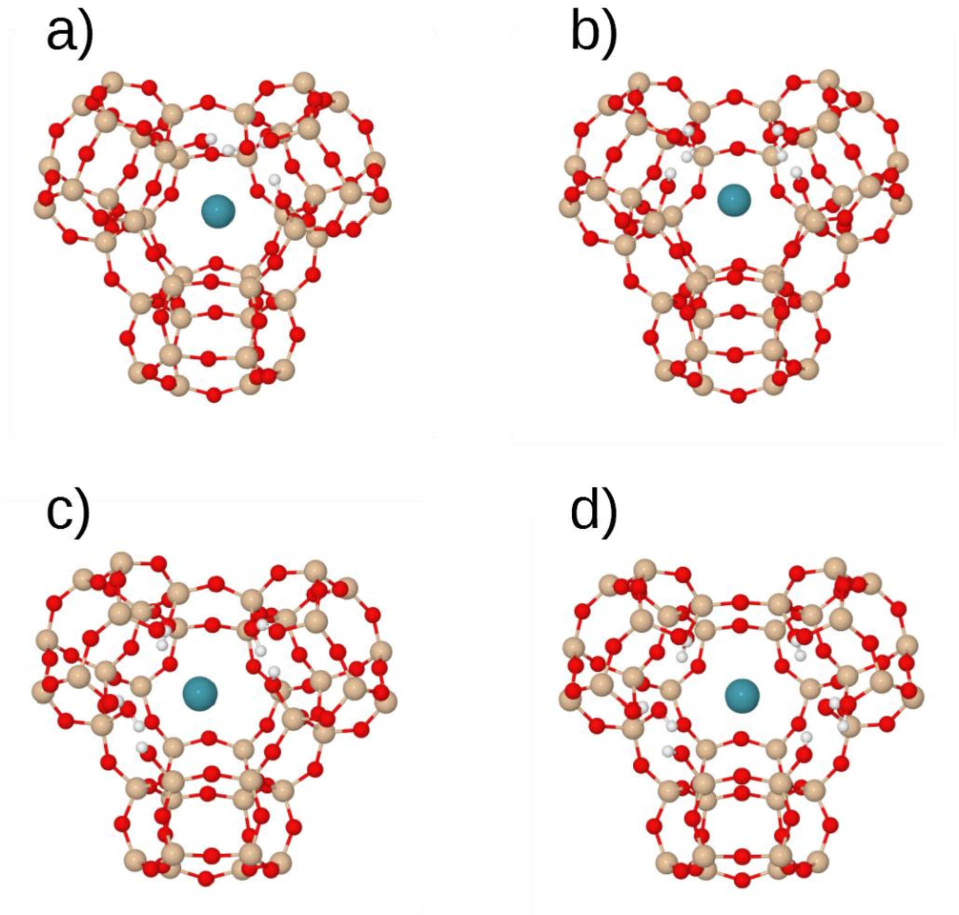

Figure S10. Four structural models of siliceous FAU with silanol nest defects located on the six membered rings separating the sodalite cage from the supercage: (a) model D: one defect, (b) model E: two defects, (c) model F: three defects, and (d) model G: four defects.

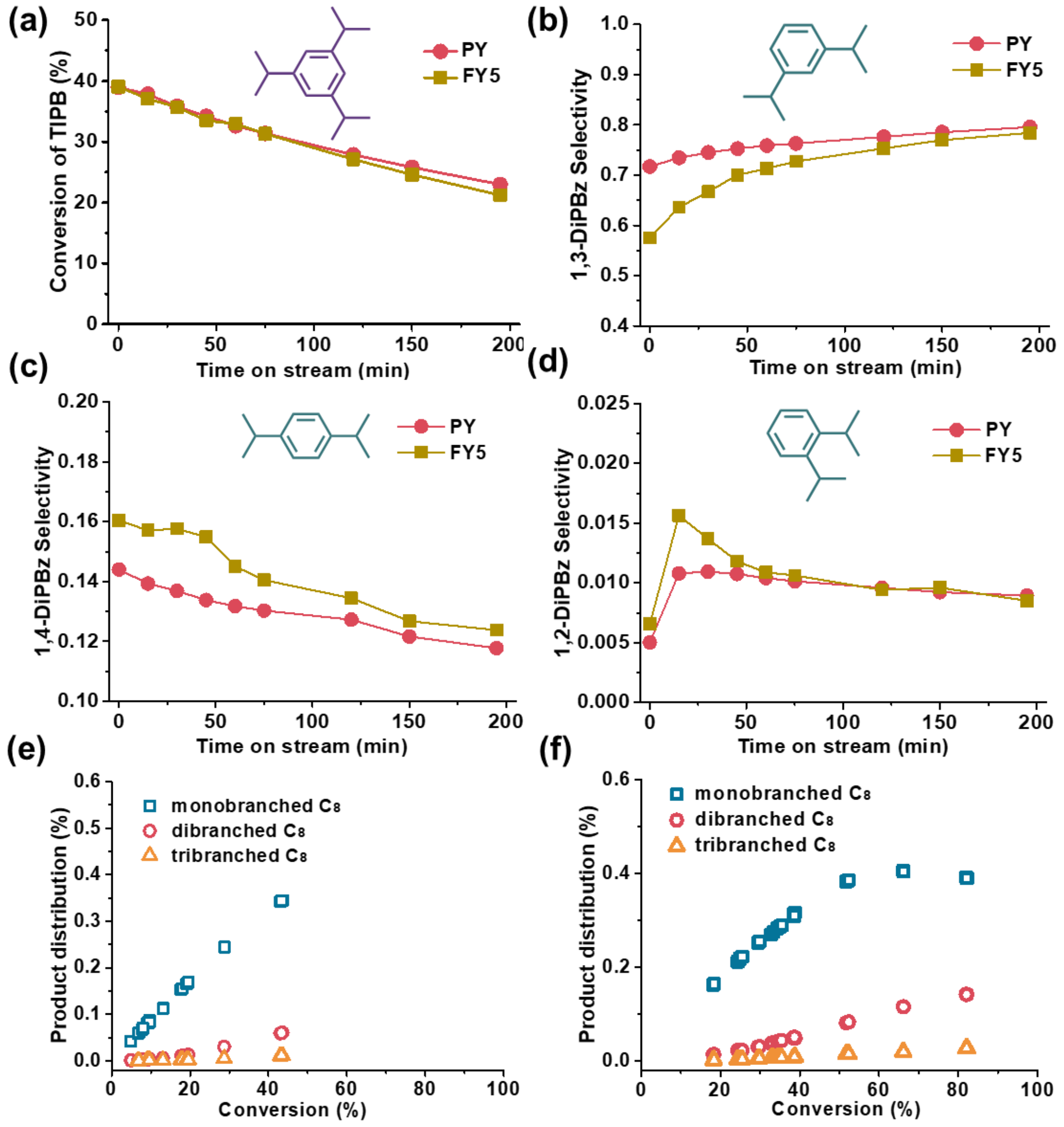

Figure S11. Dealkylation of 1,3,5-triisopropylbenzene (TIPB) and hydro-conversion of n-octane on the parent (PY) and hierarchical (FY5 and FY60) zeolites. (a) TIPB conversion as a function of time on stream (*Reaction conditions*: T = 453 K, W/F° = 0.022 kg·h·mol^{-1}, P = 0.1 MPa.[2]) (b) 1,3-diisopropylbenzene (1,3-DiPBz) selectivity, (c) 1,4-diisopropylbenzene (1,4-DiPBz) selectivity, (d) 1,2-diisopropylbenzene (1,2-DiPBz) selectivity; Products distribution vs. conversion in the hydro-conversion of n-octane (*Reaction conditions*: T = 573 K, W/F° = 8-20 kg·h·mol^{-1}, P = 5 MPa, H_2/n-C_8 = 6-500): (e) PY zeolite.[2] (f) FY60. Lines only serve to guide the eyes.

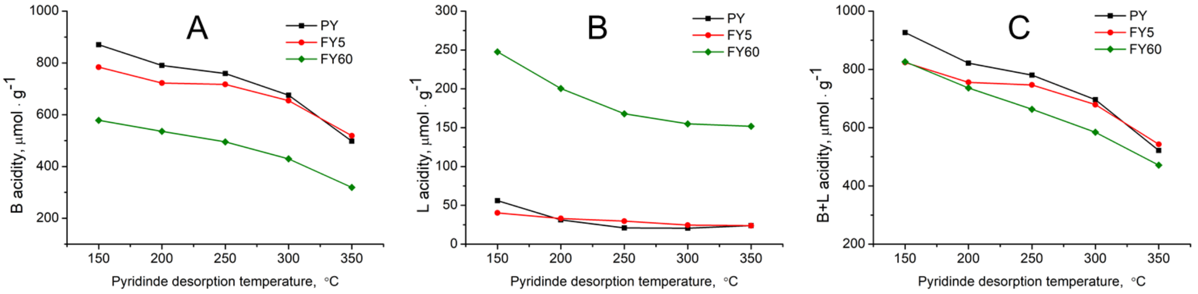

Figure S12. The comparison of the Brønsted acidity (A: B acidity), Lewis acidity (B: L acidity) and the total acidity (C: B+L acidity) of the parent and NH_4F treated zeolite samples (FY5 and FY60).

References

[1] J. Jeener, B. H. Meier, P. Bachmann, R. R. Ernst, *J. Chem. Phys.* **1979**, 71, 4546.

[2] Z. Qin, K. A. Cychosz, G. Melinte, H. Siblani, J. P. Gilson, M. Thommes, C. Fernandez, S. Mintova, O. Ersen, V. Valtchev, *J. Am. Chem. Soc.* **2017**, 139, 17273.
